# Sweet syndrome: A retrospective study of 64 cases and proposal of an algorithmic approach to improve investigation and management

**DOI:** 10.1002/ski2.23

**Published:** 2021-03-31

**Authors:** N. H. Gopee, F. G. Charlton, P. J. Hampton

**Affiliations:** ^1^ Department of Dermatology Royal Victoria Infirmary Newcastle upon Tyne UK; ^2^ Department of Pathology Royal Victoria Infirmary Newcastle upon Tyne UK

## Abstract

**Background:**

Sweet syndrome (SS) can be categorized as classical Sweet syndrome (CSS), malignancy‐associated Sweet syndrome (MASS) or drug‐induced Sweet syndrome (DISS). Appropriate categorization of patients with SS and identification of the associated trigger are essential to direct subsequent investigations and follow‐up, especially given that 21% of cases are malignancy‐associated. However, no published guidelines exist to guide this.

**Objective:**

To analyse the categorization, management and outcomes of patients with SS in order to propose a structured approach for investigation and follow‐up.

**Methods:**

Retrospective data collection from the electronic records of patients diagnosed with SS between 1 January 2005 and 31 December 2018. Categorized and non‐categorized patients were compared, and the yield rate of investigations and duration of follow‐up were analysed.

**Results:**

Sixty‐four patients were included with CSS (77%), MASS (20%) and DISS (3%). Of these, 34 (53%) cases were not categorized by the assessing clinicians, three of which were subsequently diagnosed with a malignancy, up to 19 months later. There was no significant difference in investigations performed between categorized and non‐categorized patients and the yield rates were modest overall. Follow‐up averaged 10.5 (16.8) months; non‐categorized patients were followed‐up for significantly longer than categorized patients (15.0 (21.2) vs. 5.4 (6.8) months, *p* < 0.05).

**Conclusion:**

The lack of a structured way to approach patients with SS can lead to under‐ or over‐investigation, diagnostic delays of underlying conditions and unnecessary follow‐up. An algorithm is proposed to identify the likely trigger and manage patients accordingly. Larger prospective studies are required to confirm the optimal approach to investigate and follow‐up patients with SS.

## Graphical abstract

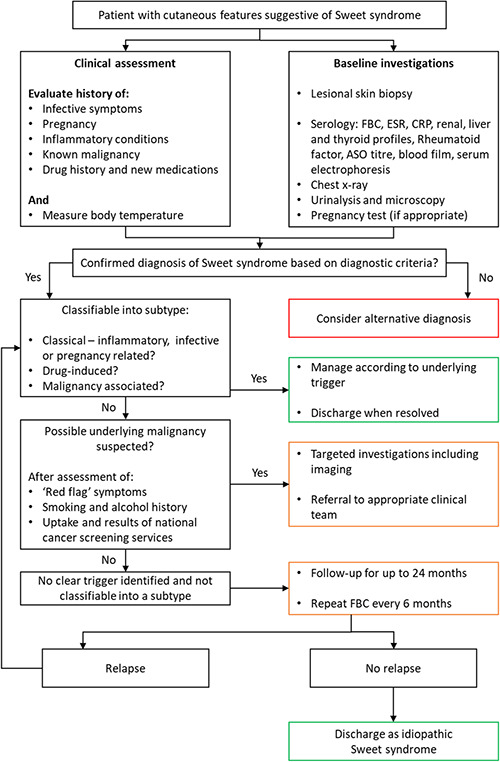

In patients with Sweet syndrome, identification of the suspected trigger and appropriate categorisation (classical, malignancy‐associated or drug‐induced Sweet syndrome) are essential to direct subsequent investigations and follow‐up, especially given that 21% of cases are malignancy‐associated. In the absence of consensus guidelines, this retrospective study analysed the categorisation, management and outcomes of patients with Sweet syndrome over a period of 13 years in order to propose a structured, algorithmic approach to prevent diagnostic delays and unnecessary investigations and follow‐up.

1

**What is already known about this topic?**

- Sweet syndrome can be triggered by several conditions, including malignancy in about 21% of cases and identifying the underlying cause is important.
- No published consensus guidelines exist to guide this.

**What does this study add?**

- This study demonstrates that inconsistencies, driven by lack of guidelines, in how patients with Sweet syndrome are categorized, investigated and followed‐up can lead to inappropriate tests, unnecessary reviews and delays in diagnosis of underlying conditions.
- An algorithm is suggested to address this which includes a stepwise identification of the likely trigger of Sweet syndrome and categorization of patients accordingly to guide subsequent investigations and follow‐up.

## 2 INTRODUCTION

Sweet syndrome (SS), or acute febrile neutrophilic dermatosis, is an inflammatory condition characterized by the abrupt onset of tender erythematous papules, nodules or plaques which demonstrate a predominantly neutrophilic infiltrate in the upper dermis on histopathological analysis.^1^ ^,^ ^2^ Three main subtypes are recognized based on aetiology: (i) classical SS (CSS) which includes cases precipitated by infection, inflammatory conditions, pregnancy and idiopathic cases where no trigger is identified, (ii) malignancy‐associated SS (MASS) and (iii) drug‐induced SS (DISS).^1^ ^,^ ^3^

The optimal management and follow‐up of patients with SS relies on identifying the likely subtype of SS, categorizing patients accordingly and performing appropriate investigations to confirm the trigger.^2^ This is particularly relevant given that 21% of cases of SS are associated with an underlying malignancy. Sweet syndrome can precede, follow or appear concurrently with the malignancy or its recurrence and can, therefore, be a cutaneous harbinger of an unsuspected cancer.^1^ ^,^ ^4^ ^,^ ^5^

Several investigations, including a malignancy work‐up, have been recommended to assess patients with SS, consisting of serological, microbiological, immunological and radiological tests.^2^ ^,^ ^5^ ^,^ ^6^ It is, however, unclear whether all patients with SS should undergo these investigations or whether a more tailored approach should be adopted. For instance, a patient with a clear infective cause may require fewer investigations compared to a patient with no identifiable trigger clinically. Clinicians' attempts to categorize patients with SS may, therefore, influence the subsequent approach to investigations and management.

To the best of our knowledge, no study has explored this to determine the optimal way to assess patients with SS to avoid under‐ or over‐investigations and prevent delays in identification of serious underlying conditions. Recent studies on SS have predominantly focused on investigating factors associated with MASS, identifying older age, anaemia, thrombocytopaenia, leucopoenia and lack of arthralgia as potential indicators of MASS.^3^ ^,^ ^7^ ^,^ ^8^ Additionally, no published guidelines exist to inform the necessary investigations and follow‐up required in these patients.

This study was conducted to analyse the approach adopted to assess and manage a cohort of patients with SS and their subsequent outcomes with the aim of proposing a structured way to investigate and follow‐up this group of patients.

## 3 PATIENTS AND METHODS

All cases recorded as SS in the clinical database and coded as SS in the histopathological database in our tertiary Dermatology centre between 1 January 2005 and 31 December 2018 were identified. Cases were included based on diagnostic criteria defined by von den Driesch^9^ for CSS and MASS and by Walker and Cohen^10^ for DISS, and required histopathological confirmation for inclusion. Patients with histiocytoid SS, characterized by predominant infiltration with immature myeloid cells on histology, were also included.^1^ ^,^ ^11^

Data on patient demographic, clinical presentation, laboratory findings, histological subtype, trigger of SS, duration of follow‐up, number of visits and outcomes was collected from the electronic medical records (Cerner Millennium) which integrates details of patients' encounters, investigations, results, letters and more recently clinical notes. We recorded whether there was an attempt to categorize patients into a subtype of SS by the diagnosing clinicians (categorized or non‐categorized). ‘Non‐categorized’ indicates that there was no clear documentation of the suspected subtype of SS in the patient's records. This retrospective study was approved by the local institutional research and development committee (Newcastle upon Tyne Hospitals research and development office and Newcastle Joint Research Office) with waiver of informed consent.

The study data were descriptively analysed in Excel (version 1912; Microsoft Office 365 ProPlus). Statistical analyses were conducted using GraphPad Prism (version 8.0; GraphPad Software.). Fisher's exact test (two‐tailed) and Mann–Whitney *U* test were used to compare the frequency of investigations and duration of follow‐up respectively between categorized and non‐categorized patients. *p* values of less than 0.05 were considered statistically significant.

## 4 RESULTS

Of the 90 records reviewed, 64 cases (43 women, 21 men) with a mean (*SD*) age of onset of 54 (18) years (range 20 months–89 years) were included. After complete examination of the medical records, 49 (77%) patients had CSS which was associated with an infection in 17 (27%) patients, inflammatory conditions in 8 (12.5%) and no identifiable trigger in 24 cases (37.5%). The female to male ratio for CSS was 2.8 to 1. Sweet syndrome was associated with a haematological disorder in 11 cases (17%), solid organ malignancy in 2 (3%) and was drug‐induced in 2 (3%). Overall, MASS accounted for 20% of cases in our cohort, with a ratio of haematological to solid organ malignancy of 85% to 15%, consistent with reported figures in SS.^4^ ^,^ ^5^ Ten patients were diagnosed with histiocytoid SS, six (60%) of which had MASS of haematological origin.

**FIGURE 1:**
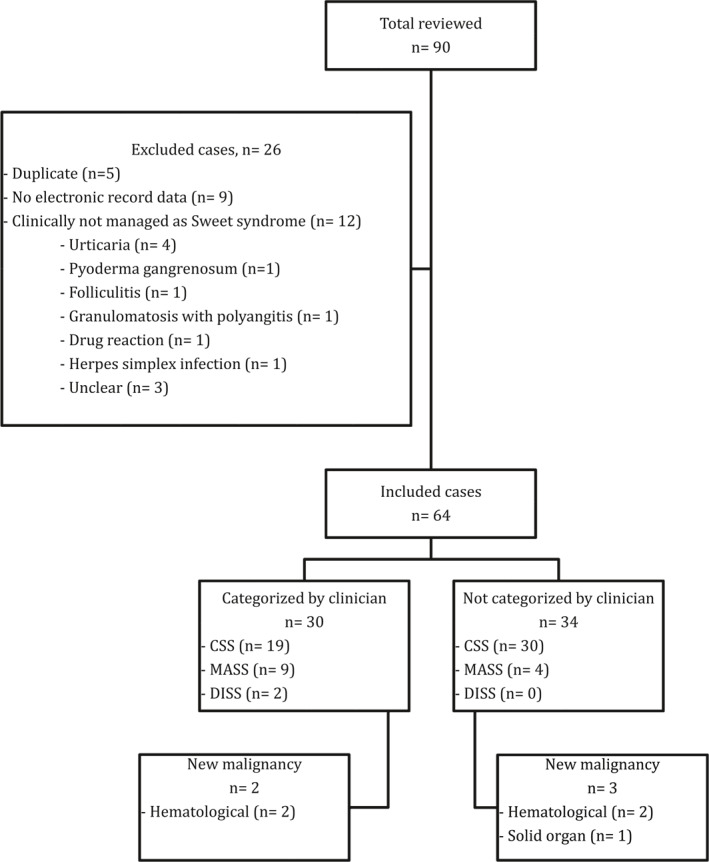
Summary flowchart of the number of cases included, categorized by the assessing clinicians and new diagnoses of malignancy. CSS, classical Sweet syndrome; DISS, drug‐induced Sweet syndrome; MASS, malignancy‐associated Sweet syndrome

In 34 cases (53%), there was no attempt made by the diagnosing clinician to categorize patients based on the suspected trigger, nine of whom had an identifiable cause at presentation–infection (5), inflammatory condition (3) or haematological malignancy (1) (Figure 1). Four cases were later classed as infection‐related when they relapsed with a concurrent infection. Three patients were subsequently diagnosed with a malignancy thought to be associated with their SS: two patients were diagnosed with myelodysplastic syndrome at 8 and 19 months after their initial presentation and one patient presented with a colonic tumour 1 week after their diagnosis of SS (Figure 1).

The frequency of the different investigations undertaken, overall and divided according to patient categorization, and the yield rates are shown in Table 1. Of 22 patients having a computed tomography (CT) scan, six had pathological findings. Four of these (sarcoidosis, bronchiectasis, colon cancer and metastatic bladder cancer) were considered contributory to the patients' SS. Six patients had bone marrow trephine biopsies which identified three cases of myelodysplastic syndromes, one patient with soft dysplastic features and one with possible myeloma. There was no significant difference in the frequency of individual investigations carried out in categorized and non‐categorized patients, except for serum electrophoresis, which was requested more frequently in non‐categorized patients compared to categorized patients (56% vs. 10%, *p* = 0.0002) (Table 1).

**TABLE 1:** Frequency of investigations performed in patients with Sweet syndrome, rate of abnormal results and comparison between categorized and non‐categorized patients

Types of investigations	Investigations overall		Investigations by subgroup		
	Total number	Abnormal results (%)	Number in categorised patients (%)	Number in non‐categorized patients (%)	*p*
*Serological*
Blood counts and acute phase reactants			28 (93.3)	30 (88.2)	0.676
Leucocytosis	58	19 (32.8)			
Neutrophilia	58	25 (43.1)			
Leukopenia	58	9 (15.5)			
Anaemia	58	25 (43.1)			
Thrombocytopaenia	57	7 (12.3)			
Raised CRP	45	41 (91.1)			
Raised ESR (>20)	37	30 (81.0)			
Blood film	18	16 (88.9)	10 (33.3)	8 (23.5)	0.417
Serum electrophoresis	22	0 (0.0)	3 (10.0)	19 (55.9)	<0.001*
Antistreptolysin O titre	8	2 (25.0)	1 (3.3)	7 (20.5)	0.058
Autoimmune screen	14	2 (14.3)	4 (13.3)	10 (29.4)	0.142
Thyroid function tests	3	0 (0.0)	0 (0.0)	3 (8.8)	0.241
Tumour markers	4	0 (0.0)	2 (6.7)	2 (5.9)	1
*Radiological*					
Chest X‐ray	8	1 (12.5)	3 (10.0)	5 (14.7)	0.713
Ultrasound abdomen	4	0 (0.0)	2 (6.7)	2 (5.9)	1
CT scan	22	6 (27.3)	12 (10.0)	10 (29.4)	0.435
Bone marrow	6	5 (83.3)	4 (13.3)	2 (5.9)	0.407
Microbiology swabs	9	0 (0.0)	2 (6.7)	7 (20.6)	0.156

Abbreviations: CRP, c‐reactive protein; CT, computed tomography; ESR, erythrocyte sedimentation rate.

*Significant at *p* < 0.05.

Fifty‐one (80%) patients were followed‐up under Dermatology care. Four patients were offered follow‐up appointments but did not attend and two patients were monitored by other clinical teams, namely haematology (*n* = 1) and neurology (*n* = 1). The average duration of follow‐up overall was 10.5 (16.8) months with 6 (6) visits per patient. Non‐categorized patients were followed up for significantly longer compared to categorized patients (15.0 (21.2) months vs. 5.4 (6.8) months respectively, *p* = 0.04).

## 5 DISCUSSION

This study analysed the approach adopted to investigate and manage an unselected sample of patients with SS over a 13‐year period. In over half of the cases, there was no potential cause suggested by the assessing clinicians and no categorization into the likely subtype of SS. Although nearly all patients had various investigations performed, the lack of attempt to classify the subtype of SS and adopt a logical approach to assessment may have led to either under‐ or over‐investigation.

A proportion of non‐categorized patients were eventually diagnosed with a malignancy—two patients, discharged without categorization or an identifiable trigger, were diagnosed with myelodysplastic syndrome when their SS relapsed up to 19 months after the initial presentation with delayed bone marrow biopsies. This indicates that proper categorization of patients at the time of initial diagnosis with directed investigations and appropriate follow‐up are required for patients with no identifiable cause of SS.

Conversely, comparison of investigations rates in categorized and non‐categorized patients suggests the potential for over‐investigation in the absence of clear guidance. There was no significant difference in how categorized and non‐categorized patients were investigated, except for serum electrophoresis (Table 1). This is further reflected in the modest yield rates of investigations overall. The highest pick‐up rate was observed for bone marrow sampling guided by blood counts and blood films. However, it is worth noting that three out of six (50%) pathological CT findings were identified through apparent blind scanning of patients which highlights the need for a careful and thorough clinical evaluation of patients with SS.

Establishing the likely underlying trigger in SS is a key step to direct further investigations, treatment and follow‐up.^2^ This is particularly important in cases of SS associated with inflammatory conditions, MASS and DISS to avoid delays in diagnosis and treatment of associated conditions and necessary drug‐withdrawal.^2^ However, the optimal way to assess these patients is not clearly defined. Recommended investigations include a lesional skin biopsy and serological tests (blood counts, acute phase reactants, hepatic and renal profiles, antistreptolysin O titre, rheumatoid factor and thyroid function).^1^ ^,^ ^2^ ^,^ ^6^ Additional investigations, such as brain and chest imaging, electroencephalograms and cerebrospinal fluid analysis, may be required to assess for extra‐cutaneous SS guided by site‐specific symptoms and suspected organ involvement.^1^ ^,^ ^2^ ^,^ ^6^ Cohen and Kurzrock^5^ also proposed a malignancy work‐up for patients with SS and no known cancer.^5^ ^,^ ^12^ They recommended additionally undertaking thyroid and lymph node examination, gynaecological (breast, cervical, uterine and ovarian) assessment in women, prostate and testicular examination in men, carcinoembryonic antigen level, urinalysis and urine culture, colon cancer screening and chest radiography.^5^

Routine scanning with CT or positron emission tomography‐CT has also been suggested. This is based on reports of the evaluation of malignancy in paraneoplastic syndromes which highlight that investigating such patients based solely on red‐flag symptoms and cancer screening guidelines may be not be aggressive enough.^6^ ^,^ ^13^, ^14^, ^15^ In contrast, the literature on SS suggests that aggressive investigative procedures are unwarranted, and that assessing for malignancy should be guided by reasonable clinical suspicion in the absence of other aetiological factors causing SS.^2^ ^,^ ^6^ However, SS can precede the development of malignancy.^1^ ^,^ ^3^ It is, therefore, advised that patients without an obvious cause for SS should be followed‐up, for at least 16 months in some reports,3 with repeat periodic testing of blood counts (6–12 monthly) to exclude a possible underlying haematological malignancy.^1^ ^,^ ^5^

**FIGURE 2:**
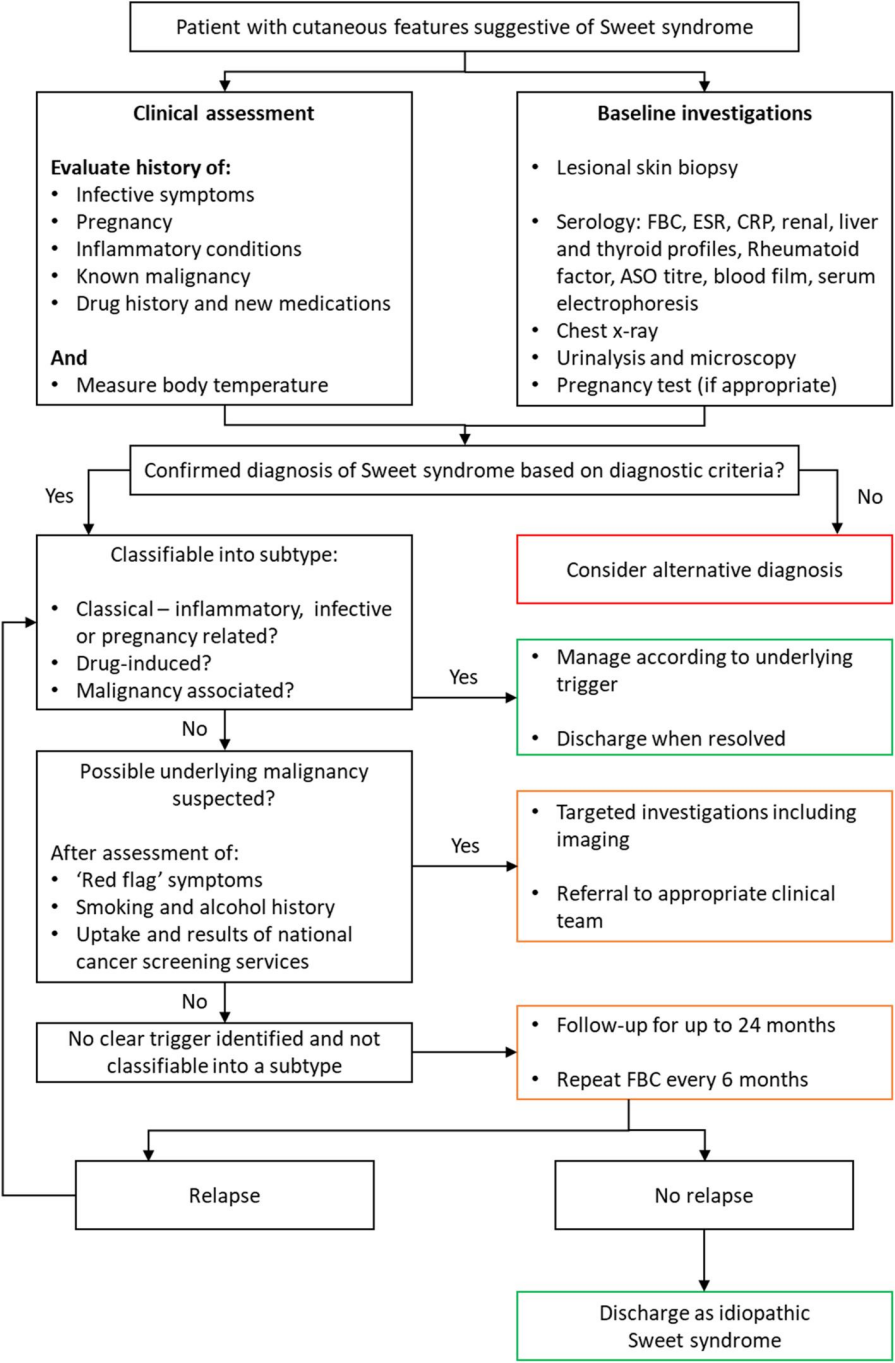
Proposed flowchart to investigate patients with cutaneous Sweet syndrome. ASO, antistreptolysin O; CRP, c‐reactive protein; ESR, erythrocyte sedimentation rate; FBC, full blood count

The findings of this study emphasize the need to adopt a structured approach to the assessment and management of patients with SS to avoid under‐ or over‐investigations and delays in diagnosis. We suggest that assessment should involve a stepwise identification of the likely aetiological factor and categorization of patients accordingly and an algorithm is proposed to support this (Figure 2). Assessment should include a meticulous evaluation for infective or inflammatory conditions, pregnancy and potential drug triggers. The identification of a clear precipitating factor can reduce the need for excessive investigations. In the absence of a definite trigger, a search for malignancy should be considered which may include further serological tests and targeted investigations, based on clinical signs and symptoms, and ensuring up‐to‐date uptake of national cancer screening tests. If a clear cause remains unidentifiable, there is a paucity of information regarding the extent of investigations to perform. It would, however, appear prudent to follow‐up this subgroup of patients for at least 18–24 months for evolving haematological disorders.

The proposed algorithm relies on clinical assessment and currently accessible investigations. Emerging insights into the pathogenesis of neutrophilic dermatoses, of which SS is a prototypical disease, suggest their pathophysiological overlap with auto‐inflammatory diseases, the dysfunctional activation of the inflammasome and implication of several genetic mutations and inflammatory signalling molecules which could potentially be used as biomarkers to further refine the above algorithm.^16^, ^17^, ^18^ For instance, immune mediators such as IL‐1*α*, IL‐1*β* and interferon‐γ, have been found to be elevated in the dermis and serum of patients with SS.^18^ However, a precise correlation between specific immune mediators and subtype of SS has not yet been established. Genetic mutations present in auto‐inflammatory syndromes, such as MEFV in Familial Mediterranean Fever, have also been identified in neutrophilic dermatoses, with mutations in PTPN6 (protein tyrosine phosphatase non‐receptor type 6) and FLT‐3 (fms‐like tyrosine kinase 3) thought to be specifically linked to MASS, especially of haematological origin.^17^ ^,^ ^18^ These represent promising markers which could supplement our proposed algorithm in the future to further tailor the investigation and management for personalized care of patients with SS.

This study is limited by its retrospective design and the relatively small sample size from a single centre. The inconsistency in investigations undertaken across the cohort of patients also precludes definite conclusions from being drawn from the yield rates and the evaluation of their predictive potential. However, this is, to our knowledge, the first study in SS focussing specifically on disease categorization and investigations and which attempts to evaluate the most appropriate way to assess patients with SS.

Our study has shown that the lack of a logical approach to categorize and investigate patients with Sweet syndrome can lead to both under‐ and over‐investigation and delayed diagnoses of malignancy. Larger, multi‐centred studies, which prospectively assess how patients with SS are categorized, investigated and followed‐up, as well as evaluate the investigation findings and patient outcomes, are warranted. These would help to conclusively determine the optimal approach to investigate and follow‐up this group of patients and to formulate evidence‐based guidelines.

## CONFLICT OF INTERESTS

The authors declare that there are no conflict of interests.

## References

[1] Cohen PR. Sweet's syndrome—a comprehensive review of an acute febrile neutrophilic dermatosis. Orphanet J Rare Dis. 2007;2:34. 17655751 10.1186/1750-1172-2-34 PMC1963326

[2] Villarreal‐Villarreal CD , Ocampo‐Candiani J , Villarreal‐Martínez A. Sweet syndrome: a review and update. Actas Dermosifiliogr. 2016;107:369–78. 26826881 10.1016/j.ad.2015.12.001

[3] Marcoval J , Martín‐Callizo C , Valentí‐Medina F , Bonfill‐Ortí M , Martínez‐Molina L. Sweet syndrome: long‐term follow‐up of 138 patients. Clin Exp Dermatol. 2016;41:741–6. 27663147 10.1111/ced.12899

[4] Raza S , Kirkland RS , Patel AA , Shortridge JR , Freter C. Insight into Sweet's syndrome and associated‐malignancy: a review of the current literature. Int J Oncol. 2013;42:1516–1522. 23546524 10.3892/ijo.2013.1874

[5] Cohen PR , Kurzrock R. Sweet's syndrome and cancer. Clin Dermatol. 1993;11:149–57. 8339190 10.1016/0738-081x(93)90112-p

[6] Merola J. Sweet syndrome (acute febrile neutrophilic dermatosis): Pathogenesis, clinical manifestations, and diagnosis. https://www.uptodate.com/contents/sweet‐syndrome‐acute‐febrile‐neutrophilic‐dermatosis‐pathogenesis‐clinical‐manifestations‐and‐diagnosis. Accessed 31 Dec 2019.

[7] Nelson CA , Noe MH , McMahon CM , Gowda A , Wu B , Ashchyan HJ , et al. Sweet syndrome in patients with and without malignancy: a retrospective analysis of 83 patients from a tertiary academic referral center. J Am Acad Dermatol. 2018;78:303–9. 29107342 10.1016/j.jaad.2017.09.013

[8] Rochet NM , Chavan RN , Cappel MA , Wada DA , Gibson LE. Sweet syndrome: clinical presentation, associations, and response to treatment in 77 patients. J Am Acad Dermatol. 2013;69:557–64. 23891394 10.1016/j.jaad.2013.06.023

[9] von den Driesch P. Sweet's syndrome (acute febrile neutrophilic dermatosis). J Am Acad Dermatol. 1994;31:535–56. 8089280 10.1016/s0190-9622(94)70215-2

[10] Walker DC , Cohen PR. Trimethoprim‐sulfamethoxazole‐associated acute febrile neutrophilic dermatosis: case report and review of drug‐induced Sweet's syndrome. J Am Acad Dermatol. 1996;34:918–23. 8621829 10.1016/s0190-9622(96)90080-8

[11] Neoh CY , Tan AWH , Ng SK. Sweet's syndrome: a spectrum of unusual clinical presentations and associations. Br J Dermatol. 2007;156:480–5. 17300237 10.1111/j.1365-2133.2006.07677.x

[12] American Cancer Society . Guidelines for the early detection of cancer. https://www.cancer.org/healthy/find‐cancer‐early/cancer‐screening‐guidelines/american‐cancer‐society‐guidelines‐for‐the‐early‐detection‐of‐cancer.html. Accessed 31 Dec 2019.

[13] Pelosof LC , Gerber DE. Paraneoplastic syndromes: an approach to diagnosis and treatment. Mayo Clin Proc. 2010;85:838–54. 20810794 10.4065/mcp.2010.0099 PMC2931619

[14] Leatham H , Schadt C , Chisolm S , Fretwell D , Chung L , Callen PJ , et al. Evidence supports blind screening for internal malignancy in dermatomyositis: data from 2 large US dermatology cohorts. Medicine. 2018;97:e9639. 29480875 10.1097/MD.0000000000009639 PMC5943873

[15] Sparsa A , Liozon E , Herrmann F , Ly K , Lebrun V , Soria P , et al. Routine vs extensive malignancy search for adult dermatomyositis and polymyositis: a study of 40 patients. Arch Dermatol. 2002;138:885–90. 12071815 10.1001/archderm.138.7.885

[16] Marzano AV , Damiani G , Ceccherini I , Berti E , Gattorno M , Cugno M. Autoinflammation in pyoderma gangrenosum and its syndromic form (pyoderma gangrenosum, acne and suppurative hidradenitis). Br J Dermatol. 2017;176(6):1588–98. 27943240 10.1111/bjd.15226

[17] Marzano AV , Damiani G , Genovese G , Gattorno M. A dermatologic perspective on autoinflammatory diseases. Clin Exp Rheumatol. 2018;36((suppl 110)(1)):32–8. 29742056

[18] Heath MS , Ortega‐Loayza AG. Insights into the pathogenesis of Sweet's syndrome. Front Immunol. 2019;10:414. 30930894 10.3389/fimmu.2019.00414 PMC6424218

